# Cytogenetic and molecular identification of novel wheat-*Elymus sibiricus* addition lines with resistance to leaf rust and the presence of leaf pubescence trait

**DOI:** 10.3389/fpls.2024.1482211

**Published:** 2024-11-12

**Authors:** Ivan I. Motsnyi, Oleksii V. Halaiev, Tetiana G. Alіeksіeіeva, Galyna O. Chebotar, Sabina V. Chebotar, Alexander Betekhtin, Robert Hasterok, Rita Armonienė, Mahbubjon Rahmatov

**Affiliations:** ^1^ Department of General and Molecular Genetics, Plant Breeding and Genetics Institute – National Center of Seed and Cultivar Investigation, Odesa, Ukraine; ^2^ Department of Molecular Biology, Biochemistry and Genetics, Odesa I.I. Mechnikov National University, Odesa, Ukraine; ^3^ Plant Cytogenetics and Molecular Biology Group, Institute of Biology, Biotechnology and Environmental Protection, Faculty of Natural Sciences, University of Silesia in Katowice, Katowice, Poland; ^4^ Institute of Agriculture, Lithuanian Research Centre for Agriculture and Forestry, Akademija, Lithuania; ^5^ Department of Plant Breeding, Swedish University of Agricultural Sciences, Alnarp, Sweden

**Keywords:** genomic *in situ* hybridization, alien introgression, leaf hairiness, molecular markers, resistance genes, *Triticum aestivum*, wild relatives

## Abstract

**Introduction:**

Emerging new races of leaf rust (*Puccinia triticina* Eriks) are threatening global wheat (*Triticum aestivum* L.) production. Identifying additional resistance genes from all available gene pools is crucial to expanding wheat resistance to these virulent leaf rust races. Siberian wild rye (*Elymus sibiricus* L.) possesses numerous beneficial traits that can be valuable in wheat improvement. Three new wheat-*E. sibiricus* addition lines, O27-2 (BC_8_), O27-3 (BC_12_) and O193-3 (BC_12_), were developed through a backcrossing scheme in this study, using leaf rust field evaluations, molecular marker assays and cytogenetic analysis.

**Methods:**

These three lines were derived from progeny of the bread wheat cultivar ‘Obriy’ (2*n* = 6*x* = 42, AABBDD) and partial octoploid amphiploid wheat-*E. sibiricus* (2*n* = 8*x* = 56, AABBDDS^t^S^t^).

**Results and discussion:**

The lines (O27-2, O27-3 and O193-3) demonstrated strong specific leaf pubescence (hairiness) and resistance at the adult stage to a local population of leaf rust races. The response to leaf rust in these three lines significantly differed from that of the *Lr24* gene, providing evidence for a distinct resistance mechanism associated with the 3S^t^ chromosome. This study is the first to report the transfer of an *E. sibiricus* chromosome into wheat that confers leaf rust resistance. Molecular marker analysis and genomic *in situ* hybridization confirmed that lines O27-2, O27-3 and O193-3 each possess one pair of *E. sibiricus* 3S^t^ chromosomes. The resistance gene was determined to be on the additional alien chromosome in these lines. Molecular markers (*Xwmc221, Lr29F18, Sr24/Lr24*) confirmed that the lines O27-2, O27-3, and O193-3 each contain a pair of *E. sibiricus* 3S^t^ chromosomes carrying leaf rust resistance genes. These findings demonstrate that the *E. sibiricus* 3S^t^ chromosome carries the leaf rust resistance gene and that the O27-2, O27-3, and O193-3 lines can serve as novel germplasm sources for introducing this resistance into wheat breeding programs. This study contributes to broadening the genetic diversity of resistance genes available for combating leaf rust in wheat.

## Introduction

Bread wheat (*Triticum aestivum* L., 2*n* = 6*x* = 42, AABBDD) is one of the most important food crops, serving as a daily staple for human consumption worldwide (Pont et al., 2019; Shiferaw et al., 2013). According to the Food and Agriculture Organization (FAO), a 60% increase in food production is needed to feed a world population exceeding 10 billion by 2050 (Feeding the World Sustainably | United Nations). To meet this escalating demand for global food security, wheat yields need to show an annual 2% increase (Hickey et al., 2019). Unfortunately, wheat diseases are actually reducing the global wheat harvest by approximately 20% per year (Savary et al., 2019).

The genetic variation in bread wheat germplasm is limited, making it challenging to develop high-yielding, quality grain-producing and disease-resistant wheat varieties (Sansaloni et al., 2020). To harness genetic diversity in wheat breeding programs, it is necessary to access genetic diversity in wild relatives of wheat harboring beneficial traits that can contribute to wheat improvement (Molnár-Láng et al., 2015). This strategy involves introgression of novel genes from *Aegilops* spp., *Elymus* ssp., *Secale cereale* etc., into wheat, aiming to enhance productivity and stability (Johansson et al., 2020; Motsnyi et al., 2016; Simonenko et al., 1998). Hexaploid bread wheat was derived through spontaneous interspecific crosses between *T. urartu* (2*n* = 2*x* = 14, AA), *Aegilops* sp. (2*n* = 2*x* = 14, BB) and *Ae. tauschii* (2*n* = 2*x* = 14, DD) (Appels et al., 2024). As a result, homoeologous transfer of valuable genes from secondary (*Aegilops* spp.) and tertiary gene pools is challenging due to the presence of the *Ph1* and *Ph2* genes, which inhibit homoeologous pairing (Mello-Sampayo, 1971; Riley and Chapman, 1958; Sears, 1956). The *ZIP4* gene located at the *Ph1* locus has been identified as a key gene in controlling crossover recombination, preventing homoeologous chromosome pairing during meiosis, and restricting gene transfer from wild relatives (Rey et al., 2017).

Introgression hybridization is a technique for transferring genetic information from wild species to wheat, thereby increasing its genetic diversity, especially in terms of rare traits (Jiang et al., 1994). This method enables addition of individual alien chromosomes to the wheat genome, leading to the development of novel genetic resources (Wang et al., 2010). New sources of disease resistance have been identified in wild relatives of wheat (e.g. *Aegilops* spp., *Elymus* ssp., *S*. *cereale*, *Thinopyrum* spp. etc.), and resistance genes from these species have been successfully transferred into wheat, providing broad-spectrum resistance. Leaf rust, caused by *Puccinia triticina* Erikss., is one of the most prevalent wheat diseases globally, and severe outbreaks can lead to wheat yield losses of over 40% (Knott, 2012; Kolmer et al., 2013). More than 80 wheat leaf rust resistance genes have been identified in wheat and its relatives, and more than half of these genes originate from wild relatives such as *Aegilops* spp., *S. cereale*, *Elytrigia* spp., *Elymus* spp. etc (McIntosh et al., 2020). Many important genes have been successfully transferred from wild relatives to wheat, such as *Sr24*/*Lr24* from *Th. elongatum*, *Lr38* from *Th*. *intermedium*, *Lr55* from *E. trachycaulis*, *Fhb3* from *Leymus racemosus* and *Fhb6* from *E. tsukushiensis* (Cainong et al., 2015; Friebe et al., 1996; McIntosh et al., 2020; Qi et al., 2008). Genetic diversity from *Elymus sibiricus* has also been successfully transferred into the wheat genome (Motsnyi et al., 2016; Motsnyi and Kulbida, 2018).

Siberian wild rye (*Elymus sibiricus* L., 2*n* = 2*x* = 28, S^t^S^t^HH) is an important source of new genetic diversity for wheat improvement (Klebesadel, 1969; Tsvelev, 1976). This crop exhibits high resilience, is distinguished by excellent palatability and adaptability, and shows outstanding tolerance to salinity, drought and cold growing conditions (Zhang et al., 2016). *E. sibiricus* also serves as a source of resistance to various wheat fungal and virus diseases (Braverman, 1986). This grass species is a promising gene pool source for wheat improvement and could greatly contribute to wheat breeding. However, it has rarely been hybridized with wheat, suggesting that there is significant untapped potential in this area (Wang, 2011). Scientists at the Plant Breeding and Genetics Institute-National Centre for Seed and Cultivar Investigation (PBGI-NCSCI) in Odesa, Ukraine, have developed an amphiploid line combining wheat and *E*. *sibiricus* genomes (Simonenko et al., 1998). The underlying aim was to enhance wheat-breeding programs by exploiting the unique genetic characteristics of *E*. *sibiricus*. Recent developments have resulted in alien disomic addition lines obtained by crossing the wheat cultivar Obriy with the amphiploid wheat-*E*. *sibiricus* line (Motsnyi and Kulbida, 2018). This represents a significant milestone in plant genetics, illustrating the potential of innovative combination of diverse genetic attributes to produce enhanced wheat varieties. The aim of the present study was to identify the alien addition in these lines, assess their cytological stability and investigate how this addition affects wheat disease resistance. The focus was on understanding genetic integration and its practical implications for enhancing wheat disease resistance.

## Materials and methods

### Plant materials

An amphiploid (2*n* = 8*x* = 56) wheat-*E. sibiricus* was provided by Dr. R. Franke from the Institute of Breeding Research, Quedlinburg, Germany (personal communication, 2024). *E. sibiricus* (genomic composition 2*n* = 2*x* = 28, S^t^S^t^HH) was crossed with bread wheat (genomic composition 2*n* = 6*x* = 42, AABBDD) to produce the partial amphiploid (2*n* = 8*x* = 56, AABBDDS^t^S^t^) *Elytricum fertile* (hereafter called EF3). Throughout the process, cross-pollination was controlled, and careful screening and selection were performed to ensure the successful integration of genetic material from both parents. Genomic *in situ* hybridization (GISH) was conducted at the Eastern Cereal and Oilseed Research Centre, Agriculture and Agri-Food Canada, where the genomic composition of the amphiploid was determined to be 2*n* = 8*x* = 56, AABBDDS^t^S^t^ (Dr. G. Fedak, pers. comm. 2024). The SLU-9702 accession of *E*. *sibiricus* (2*n* = 4*x* = 28, S^t^S^t^HH) provided by Dr. A.V. Agafonov of the Central Botanic Garden of SB RAS, Novosibirsk, Russia, was used.

**Figure 1:**
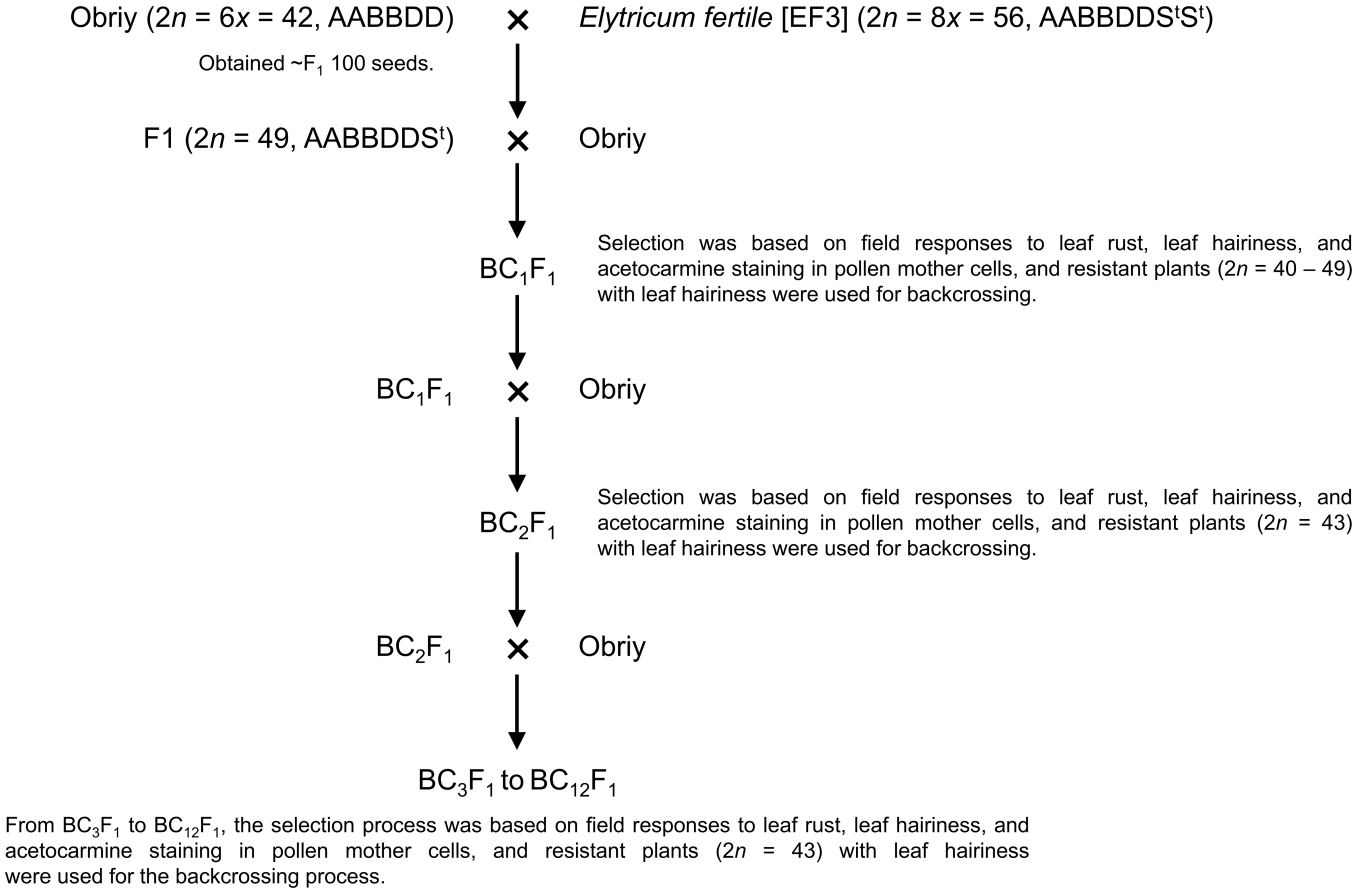
Backcrossing scheme for developing the wheat-*E*. *sibiricus* addition line O27-3 (2*n* = 44).

*Elytricum fertile* (EF3) was crossed with the cultivar Obriy, resulting in ~100 F_1_ seeds (**Figure 1**). These F_1_ plants were then self-pollinated or backcrossed to Obriy, producing about 170 F_2_ and 30 BC_1_F_1_ seeds. A total of 132 F_2_ and 29 BC_1_F_1_ plants were assessed at the adult stage for leaf rust reaction and leaf hairiness in the field, to select resistant plants that exhibited leaf hairiness (pubescence) for further backcrossing (**Figure 1**). The backcrossing process was carried out in the field, focusing on selecting leaf rust-resistant plants that exhibited leaf hairiness, with this approach continuing until the BC_12_F_1_ generation was obtained (**Figure 1**).

### Evaluation of disease resistance and leaf pubescence in the field

From 2013 to 2024, the BC_12_F_3_ to BC_12_F_∞_ generations were grown in wide rows in the field PBGI-NCSCI. During this period, the lines were exposed each growing season to natural epidemics of leaf rust (*Puccinia triticina* Eriks.), yellow rust (*Puccinia striiformis* f. sp. *tritici*) and stem rust (*Puccinia graminis* f. sp. *tritici*). The level of natural infection by leaf rust was consistently observed every year, providing an ideal environment to evaluate resistance to this pathogen. Yellow rust epidemics occurred every 3 to 4 years throughout the study, while stem rust appeared less frequently every 4 to 5 years. We planted the highly susceptible wheat cultivars Odeska 267, Obriy, and Kuyalnik in the field nurseries for leaf rust and Michigan Amber and Odeska Polukarlicova for yellow rust and stem rust to assess natural disease pressure and monitor infection levels. These susceptible cultivars served as infection indicators, confirming the presence and spread of the pathogens for a reliable evaluation of rust resistance in the BC_12_F_3_ to BC_12_F_∞_ generations under natural infection conditions. In addition, the BC_8_F_4_ to BC_8_F_9_ and BC_12_F_4_ to BC_12_F_9_ generations were tested separately under artificial inoculation for leaf rust and stem rust, respectively. The plants were artificially inoculated with a mixture of talcum powder and urediniospores of leaf rust and stem rust using a knapsack sprayer. Artificial nurseries were mist-irrigated three times daily (morning, afternoon, and evening) to maintain a moist environment and stimulate rust development. Four susceptible wheat cultivars for leaf rust and six for stem rust were used as spreader rows, positioned perpendicular to the test plots in the main wind direction to facilitate disease spread. Disease severity was assessed at the booting, heading, and dough stages using the method described by Babayants and Babayants (2014) once the susceptible spreader plants reached full infection. The plants were consistently assessed for their resistance to three rust diseases (yellow rust, stem rust, and leaf rust), and leaf hairiness (pubescence) under both natural and artificial infection conditions. Adult plant infection responses (size and type of uredinia) (Roelfs et al., 1992; Babayants and Babayants, 2014) and leaf tissue infections by the three rust diseases (0–100%) were assessed in the field using the modified Cobb scale (Peterson et al., 1948) at Zadoks growth stages 45–60 (Zadoks et al., 1974).

The leaf rust reactions of BC_12_F_2_ populations, derived from plants with 43 chromosomes and resistant to leaf rust, were evaluated in the field, using Obriy as the susceptible control. These populations were exposed to natural infection pressure and, when the cultivar Obriy was fully infected, both the parent plants and the BC_12_F_2_ individuals were assessed for their resistance to leaf rust. Phenotypic evaluations were conducted during the heading and flowering stages (Zadoks stage 50-70). To determine leaf hairiness (pubescence), the upper (adaxial) surface of leaf blades was examined using a magnifying glass. This detailed field evaluation provided a comprehensive understanding of the resistance mechanisms and phenotypic traits in these wheat lines, contributing valuable information regarding leaf rust resistance in wheat.

### Acetocarmine staining

The cytological examination was performed using standard acetocarmine staining techniques. After fixing the anthers in Carnoy fixative (containing 99% alcohol:chloroform:glacial acetic acid in a 6:3:1 ratio) and pre-treatment with 4% iron-ammonium alum, the anthers were stained with 2% acetocarmine. The acetocarmine staining analysis was performed on generations from BC_1_F_1_ through BC_12_F_1_, BC_8_F_1_ to BC_8_F_6_, and BC_12_F_2_ to BC_12_F_6_. All hybrid plants with the *E*. *sibiricus* traits of leaf hairiness and resistance to leaf rust, as well as some plants without the *E*. *sibiricus* traits were analyzed cytologically. At each plant, at least 30 distinct plates at the meiotic metaphase 1 (M1) stage were examined in pollen mother cells (PMCs). Univalent, open and closed bivalents were treated.

### Statistical analysis

The data were analyzed using descriptive statistics and analysis of variance (ANOVA) with the STATISTICA 8 software package (Statsoft, 2005). Before statistical analysis, percentage values were converted to Fisher’s angular coefficient (*φ*) in radians to normalize the frequency distribution. A 95% confidence interval and the least significant difference at the *p*<0.05 level (LSD_0.05_) were employed to determine the differences’ significance. For comparing data expressed as percentages, confidence limits were calculated using the formula for alternative variability: *t*×*Sp*, where *t* – Student’s coefficient at *p*<0.05 = 1.96, and *Sp* – standard error for alternative variability, calculated as $\sqrt{(\%\times (100-\%))/\text{n}}$ (Fomich, 1973).

### Fluorescence *in situ* hybridization

#### Chromosome slide preparation

Mitotic cells from the 2- to 3-day-old root-tip meristems of BC_12_F_6_ (O27-2) and BC_12_F_6_ (O27-3 and O193-3) disomic lines were treated in ice-cold water for 24 hours, and used for chromosome slide preparation as described by Jenkins and Hasterok (2007). Seeds were germinated on moistened filter paper at 20-22°C in darkness until the roots reached 1.5-2 cm long. The seedlings were then fixed in a 3:1 (v/v) mixture of methanol and glacial acetic acid and stored at -20°C until needed. The excised roots were washed in 0.01 M citric acid-sodium citrate buffer (pH ~4.8) for 20 minutes and then subjected to enzymatic digestion for 1-1.5 hours at 37°C using a mixture of 1% (w/v) cellulose (Calbiochem), 1% (w/v) Onozuka R-10 cellulase (Serva) and 20% (v/v) pectinase (Sigma). After separating non-meristematic parts, the root tips were squashed in a drop of 45% acetic acid, and the preparations were rapidly frozen. Following the removal of coverslips, the slides were air-dried and stored in a refrigerator for future use.

#### DNA probes

The following probes were used: (1) For GISH, total nuclear DNA was extracted from *E*. *sibiricus* seeds (Saghai-Maroof et al., 1984) and then labeled by nick translation with digoxigenin-dUTP (Hasterok et al., 2002; Maluszynska, 2002). (2) The 25S rDNA probe was produced using nick translation with tetramethyl-rhodamine-dUTP on a 2.3 kb ClaI subclone of the 25S rDNA coding region from *Arabidopsis thaliana* (Unfried and Gruendler, 1990). This probe effectively identified the loci of the 18S-5.8S-25S rRNA genes in FISH experiments.

#### FISH procedure

The slides were initially treated with RNase (100 μg/mL) in 2×SSC at 37°C for one hour, followed by a wash in 2×SSC (saline sodium citrate) and dehydration in ethanol. The hybridization mixture for the cloned probes consisted of 50% deionized formamide, 10% dextran sulphate, 2× SSC, 0.5% SDS and approximately 3 ng/L (25-100 ng/slide) of each probe DNA. This mixture was pre-denatured at 75°C for 10 minutes before application to chromosome preparations. Slides and DNA probes were denatured at 75°C for 3 minutes in an *in situ* thermal cycler (Hybaid), and then hybridized overnight at 37°C in a humid chamber. Following stringent washing (10-20% formamide in 0.1×SSC at 42°C for 10 minutes), immunodetection of digoxigenated probes was performed using FITC-conjugated anti-digoxigenin antibodies (Roche). Finally, counterstaining and mounting were carried out using 2.5 g/mL DAPI in a Vectashield antifade buffer.

#### Image capturing and processing

Images were captured using two set-ups: an AxioCam MRm monochromatic camera (Zeiss) attached to a Zeiss AxioImager.Z.2 wide-field epifluorescence microscope and an Olympus Camedia C-4040Z digital camera connected to a Leica DMRB epifluorescence microscope. Image processing and superimposition were conducted using FIJI software. Three slides from different meristems were analyzed for each disomic line and its parental forms, including the amphiploid EF3 and *T*. *aestivum*.

### Molecular marker analysis

DNA was extracted from young leaves, and PCR analysis and fragment detection were performed using the CTAB procedure (Saghai-Maroof et al., 1984). The DNA was isolated from six individual plants from each parental form, which included *Elymus* species, EF3, and cv. Obriy, and 10 individual plants from each experimental line. We also used lines containing the *Lr34* resistance gene and *Lr* genes transferred from the S^t^ genome of *Th. ponticum* to the bread wheat genome, such as *Lr19* (7DL-7Ae#1L), *Lr24* (3DL-3Ae#1L), and *Lr29* (DL-7Ae#1L·7Ae#1S). The molecular markers *Xwmc221* for *Lr19*, *Sr24*/*Lr24#12* for *Lr24*, *Lr29F18* for *Lr29*, and *csLV34* for *Lr34* were assessed (Galaev and Sivolap, 2015; Gorash et al., 2014).

### Field evaluations of agronomic performances

Field experiments were conducted at PBGI-NCSCI. Sowing was performed using a tractor-drawn breeding seed drill, SSFK-7, with each plot covering 10 m^2^ and a seed rate of 5 million germinating grains/ha. The number of spikes per m^2^ was assessed using a 0.5 m × 0.5 m frame placed in the middle of each plot, from which 25-30 plants were randomly selected for agronomic performance analysis. These traits encompassed heading date, plant height, spike length, spikelet number, spike density, number of kernels per spike and thousand kernel weight. Grain quality was evaluated using the SDS30′K sedimentation value, as described by Rybalka et al. (2006). Protein content was determined using the Kjeldahl method with a Kjeltec-Auto 1030 instrument (Foss Electric, Denmark), while the thousand kernel weight was measured according to the DSTU 4138-2002 method.

## Results

### Population development

**Figure 2:**
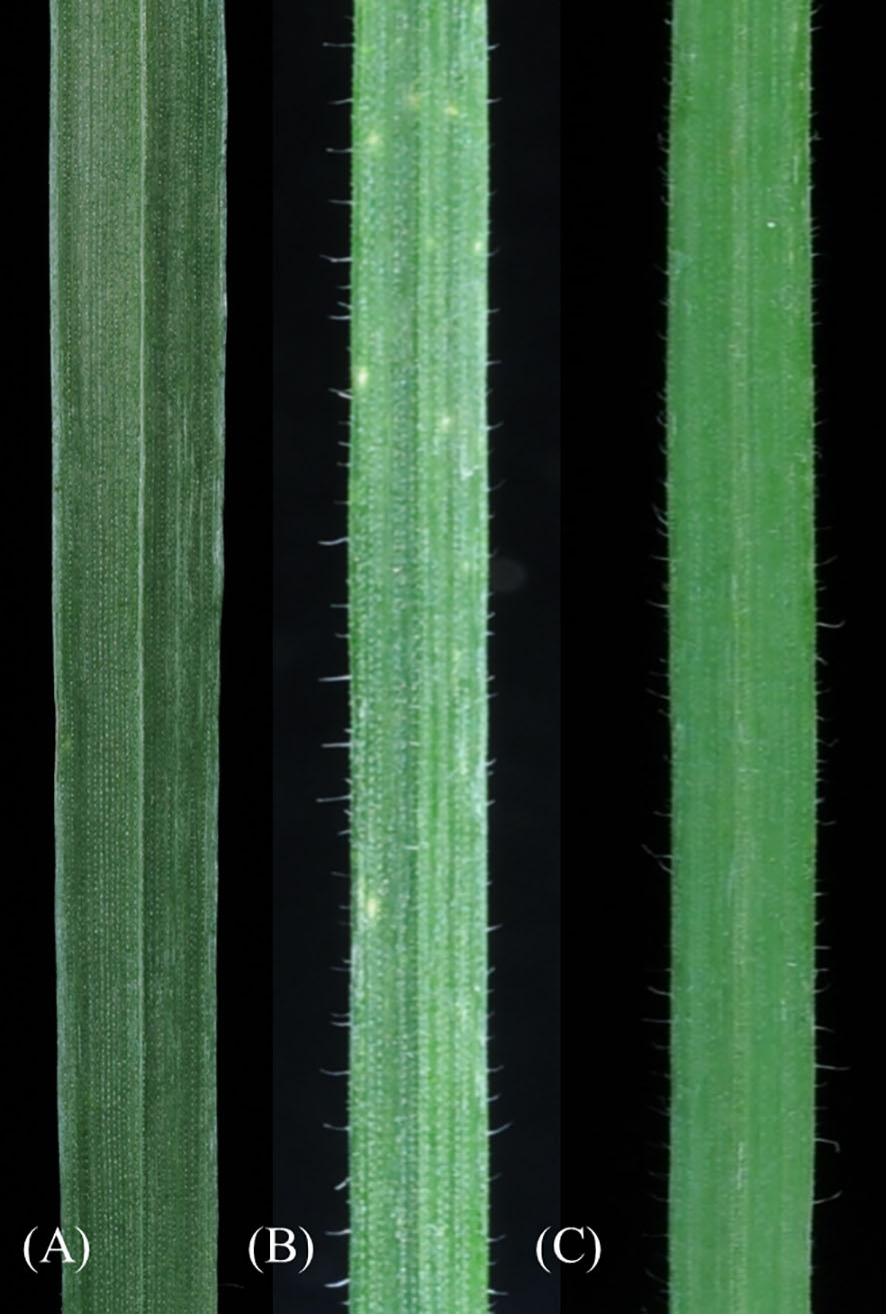
Leaf glabrous and pubescence (hairiness) morphologies. **(A)** Cultivar Obriy (2*n* = 42), showing glabrous leaves, **(B)** EF3 (2*n* = 8*x* = 56), exhibiting leaf pubescence, and **(C)** the addition line O27-3 (2*n* = 44) with leaf pubescence.

In this study, addition lines were successfully developed through a backcrossing scheme using *Elytricum fertile* (EF3), and the recurrent cultivar Obriy (**Figure 1**). Selection of lines was based on a chromosome count of *2n* = 43, determined by observing acetocarmine-stained chromosomes in pollen mother cells. Plants with a chromosome number of 2*n* = 43 (21_w_^II^+1_e_^I^), along with necrotic resistance to leaf rust and strong pubescence on the upper surface of the leaf blade, were selected through each backcross generation (BC_1_F_1_ to BC_1_F_12_). The development process involved two independent, parallel series of backcrosses initiated from two distinct second-generation plants that showed clear alien traits: 27-94 (BC_1_F_1_ Obriy/EF3//Obriy, 2*n* = 48) and 193-94 (F_2_ Obriy/EF3 self, 2*n* = 45+t). In the progeny derived from the plant 27-94, an individual plant BC_8_F_1_ with a chromosome count of 2*n* = 44; 22^II^, displaying distinct marker characteristics was identified after the eighth backcross without self-pollination. After completing 12 backcrosses (as shown in **Tables 1**, **2**) and a final round of self-pollination involving 43 chromosome plants, two more independent disomic addition lines, O27-3 and O193-3, were developed. Each of the developed lines (O27-2, O27-3, and O193-3) was found to have one pair of intact *E. sibiricus* chromosomes in addition to the bread wheat genome. All three lines exhibited leaf pubescence (hairiness) and resistance to leaf rust, whereas the wheat cultivar Obriy showed glabrous leaves and was susceptible to the disease (**Figure 2**).

**Table 1:** Frequency of plants with the S^t^ chromosome of *E. sibiricus* transmitted through female gametes in the family 27-94 offspring of hybrids Obriy/EF3//Obriy^*2-12^.

Generation	Grains		Hybrids of BC_2_F_1_-BC_12_F_1_, %		
	Total sown, seeds	Germinated, %	Winter survived	Harvested^a^	Leaf rust resistant and leaf hairiness
Obriy	60	88	5	3	0
EF3	60	75	95	93	100
BC_2_F_1_	67	31.3	47.6	23.8	20.0
BC_3_F_1_	51	58.8	86.7	80.0	25.0
BC_4_F_1_	38	78.9	86.6	80.0	20.8
BC_5_F_1_	291	75.6	98.6	95.9	28.0
BC_6_F_1_	362	83.7	5.9	3.3	40.0
BC_7_F_1_	34	67.6	91.3	91.3	28.6
BC_8_F_1_	108	76.9	100.0	98.8	13.4
BC_9_F_1_	253	73.1	40.5	24.9	19.6
BC_10_F_1_	75	90.7	100.0	91.2	14.5
BC_11_F_1_	92	69.6	93.8	89.1	7.0
BC_12_F_1_	51	51.0	76.9	76.9	20.0
∑ and *p* ^b^	1422	69.0	81.9	72.5	21.1
*φ* ± *t*∙*S_φ_* ^c^		1.960 ± 0.235	2.262 ± 0.498	2.037 ± 0.503	0.955 ± 0.137

a Percentage germination.

b Total data and percentage for all years of the study.

c Transformed percentage value obtained with Fisher`s formula (φ=2arcsin√%) ± 95% confidence limits.

**Table 2:** Frequency of plants with the S^t^ chromosome of *E. sibiricus* transmitted through female gametes in the family 193-94 offspring of backcrossed hybrids Obriy/EF3 self//Obriy^*2-12^.

Generation	Grains		Hybrids of F_2_BC_1_F_1_-BC_12_F_1_, %		
	Total sown, seeds	Germinated, %	Winter survived	Harvested^a^	Leaf rust resistant and leaf hairiness
BC_1_F_1_	81	49.4	77.5	62.5	24.0
BC_2_F_1_	103	62.1	100.0	93.8	10.0
BC_3_F_1_	122	82.0	82.0	76.0	14.5
BC_4_F_1_	288	61.8	98.3	96.6	14.0
BC_5_F_1_	209	67.9	98.5	97.1	24.1
BC_6_F_1_	261	84.7	11.8	5.8	7.7
BC_7_F_1_	105	69.5	94.5	91.7	16.4
BC_8_F_1_	36	88.9	100.0	100.0	25.0
BC_9_F_1_	227	67.4	46.4	33.3	17.6
BC_10_F_1_	100	87.0	100.0	95.4	22.9
BC_11_F_1_	90	70.0	85.7	82.5	13.5
BC_12_F_1_	53	71.7	89.5	86.8	27.3
∑ і *p* ^b^	1675	71.5	88.6	81.5	17.5
*φ* ± *t*∙*S_φ_* ^c^		2.016 ± 0.171	2.452 ± 0.444	2.251 ± 0.459	0.864 ± 0.124

a Percentage.

b Total data and percentage for all years of the study.

c Transformed percentage value obtained with Fisher`s formula (φ=2arcsin√%) ± 95% confidence limits.

### Leaf pubescence and resistance to leaf rust characteristics of addition lines

**Figure 3:**
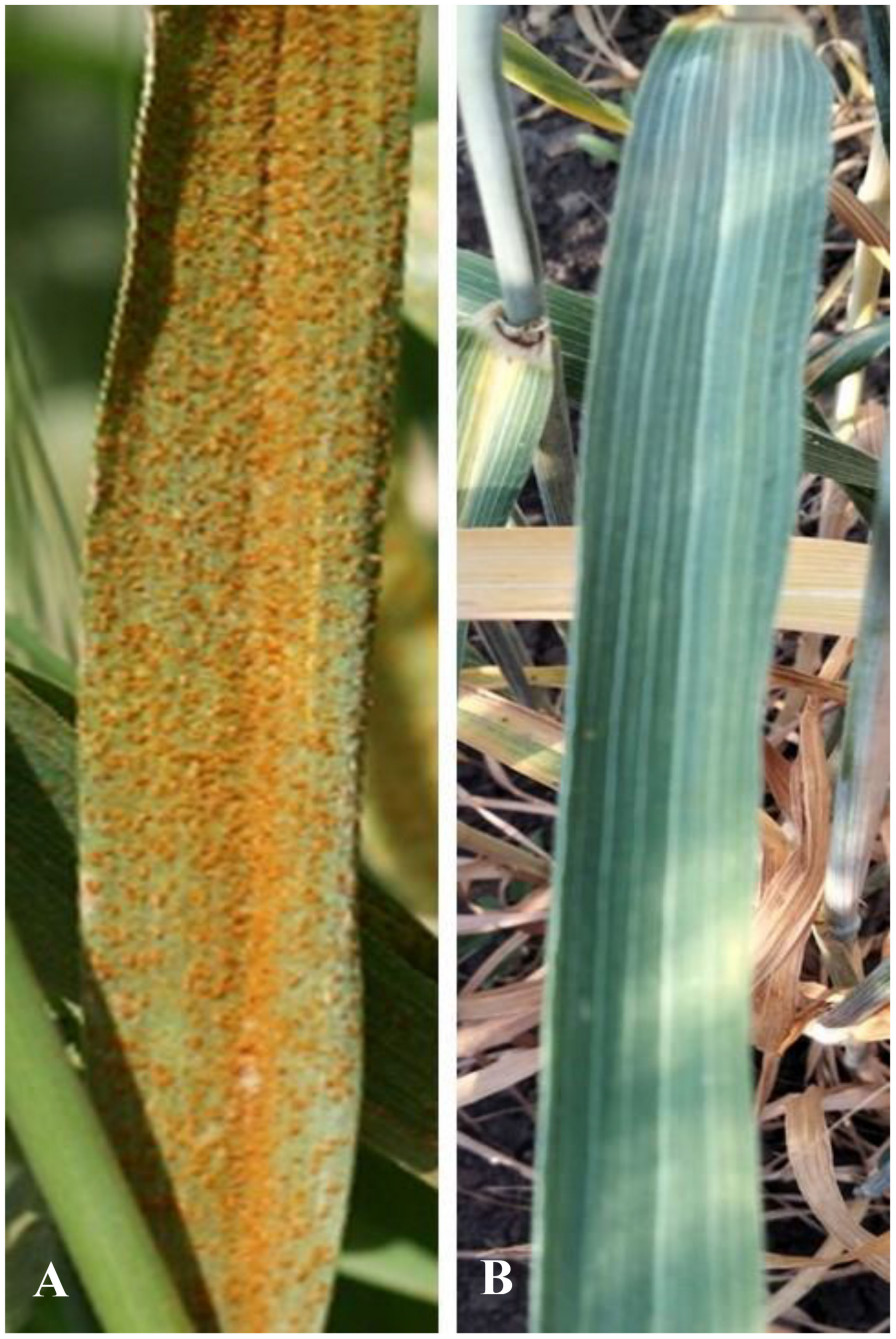
Field response to leaf rust. **(A)** Upper surface of the leaf blade of the bread wheat (*Tr. aestivum*) cultivar Obriy (2*n* = 42), showing leaf rust susceptible, and **(B)** the line О193-3 (2*n* = 44), showing resistance to leaf rust.

The EF3 amphiploid showed a spectrum of responses to leaf rust, yellow rust and stem rust, ranging from high to moderate resistance, whereas the cultivar Obriy displayed a range from moderately susceptible to very susceptible (**Table 3**). All three addition lines (O27-2, O27-3 and O193-3) showed high resistance to leaf rust, while they displayed moderate susceptibility to susceptibility to yellow rust and stem rust in field conditions (**Table 3**). The resistance responses to leaf rust were characterized by small to medium-sized necrotic flecks on wheat leaves, without any visible pustules (**Figure 3**). Recurrent wheat cultivar Obriy displayed the most severe disease symptoms, exhibiting complete susceptibility (S) to leaf rust (**Figure 3**). The addition lines and amphiploid EF3 exhibited leaf hairiness, whereas the cultivar Obriy was characterized by its lack of leaf hairiness (fully glabrous) (**Figure 2**). The observed leaf hairiness is associated with the leaf rust resistance gene identified in EF3, O27-2, O27-3 and O193-3 (**Table 3**).

**Table 3:** Resistance of the lines to rust diseases and their leaf pubescence status.

Line	Leaf rust	Yellow rust	Stem rust	Leaf pubescence	Chromosome constitution
EF3	0-5 HR-R	0-5 HR-R	5-10 R-MR	+	2*n* = 8*x* = 56, AABBDDS^t^S^t^
О27-2 (BC_8_)	0-5 HR-R	40-60 MS-S	40-60 MS-S	+	2*n* = 6*x* = 44, AABBDD3S^t^
О27-3 (BC_12_)	0-5 HR-R	40-60 MS-S	40-60 MS-S	+	2*n* = 6*x* = 44, AABBDD3S^t^
О193-3 (BC_12_)	0-5 HR-R	40-60 MS-S	40-60 MS-S	+	2*n* = 6*x* = 44, AABBDD3S^t^
Obriy	80-100 S-VS	40-60 MS-S	40-60 MS-S	–	2*n* = 6*x* = 42, AABBDD

The field response to yellow rust, leaf rust, and stem rust was assessed based on a scale of reaction (Roelfs et al., 1992) ranging from highly resistant (HR) to resistant (R), moderately resistant (MR), moderately susceptible (MS), susceptible (S) and very susceptible (VS). Leaf pubescence was assessed visually.+ Presence − or absence of the leaf hairiness.

### Location of disease resistance and leaf pubescence genes on the additional chromosome

**Figure 4:**
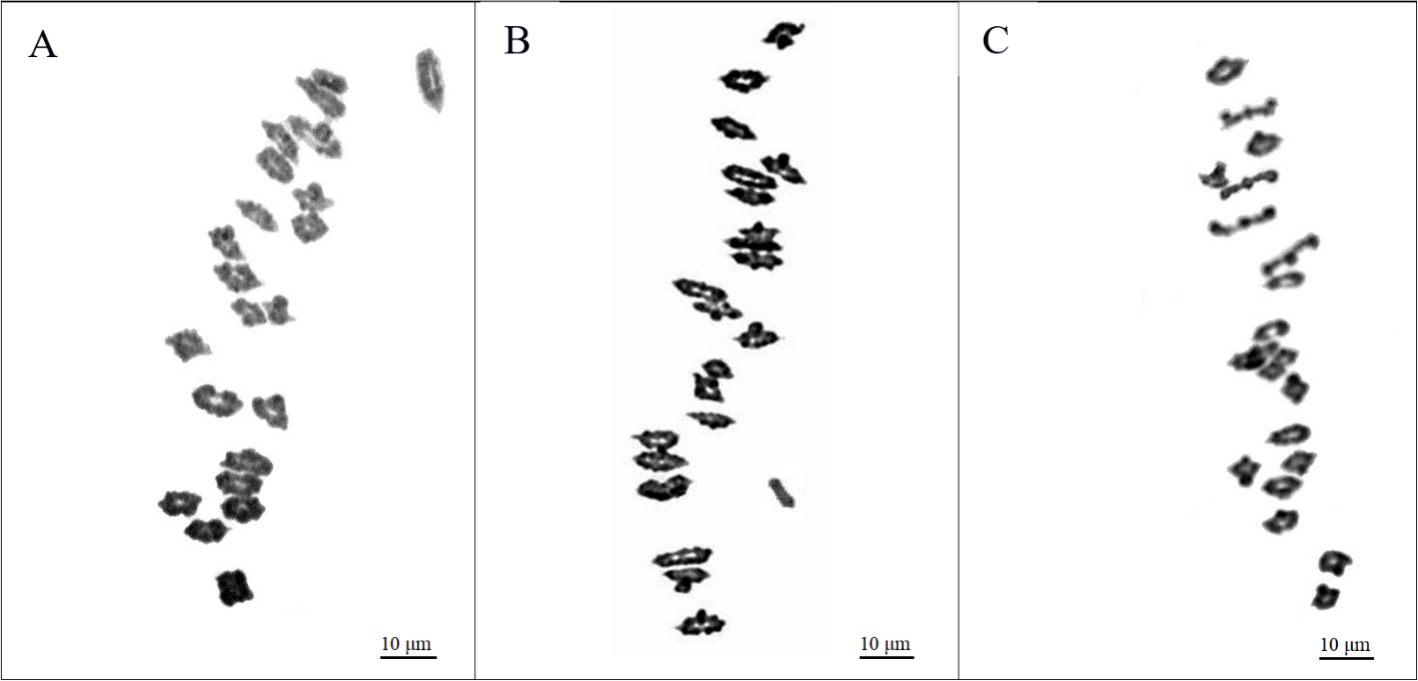
Chromosome behavior at meiotic metaphase I in pollen mother cells in typical resistant-pubescent and susceptible-glabrous plants from the ВС_12_F_2_ population of Obriy/EF3//Obriy^*12^. **(A)** Resistant-pubescent plant (2*n* = 22^II^ = 44). **(B)** Plant with 2*n* = 21^II^ + 1^I^ = 43, the univalent being an *E. sibiricus* chromosome which carries leaf rust resistance and pubescence, indicated by an arrow. **(C)** Typical susceptible individual (2*n* = 21^II^ = 42), no added *E. sibiricus* chromosome observed.

An analysis of the 27-3-94 BC_12_F_2_ segregation population, consisting of 158 individuals, revealed significant phenotypic and chromosomal differences (**Table 3**). Of these, 44 plants displayed resistance to leaf rust, and were pubescent, while the remaining 114 plants were susceptible to leaf rust, and had glabrous leaves. Meiotic analysis revealed that these groups exhibited a variety of chromosomal configurations. In the resistant-pubescent subset, two plants exhibited a chromosome count of 2*n* = 22^II^ = 44 (**Figure 4A**), whereas the remaining 42 plants had a configuration of 2*n* = 21^II^+1^I^ = 43 (**Figure 4B**). All 114 susceptible-glabrous plants consistently exhibited a chromosome configuration of 2*n* = 21^II^ = 42 (**Figure 4C**). Analysis of the BC_12_F_2_ population of 193-3-94, comprising 385 individuals, provided a number of additional insights. A total of 71 plants containing marker characters had a chromosome set of 2*n* = 21^II^+1^I^ = 43. Interestingly, only one plant was identified as a disomic addition with 2*n* = 22^II^ = 44 chromosomes, while 313 plants without alien characters were confirmed to have 42 chromosomes (**Table 4**). The addition chromosome was not observed to pair with wheat chromosomes, based on the absence of multivalents. All resistant-pubescent plants possessed one or two *E*. *sibiricus* chromosomes, whereas none of the susceptible-glabrous plants possessed any *E*. *sibiricus* chromosomes. The correlation between leaf rust resistance and pubescence was determined by analyzing phenotypic and chromosomal differences in the segregated population. Meiotic observations revealed that the genes responsible for resistance and hairiness in lines O27-2, O27-3, and O193-3 are located on an additional S^t^ chromosome from *E*. *sibiricus*. These characteristics were validated in the analysis as potential phenotypic markers.

**Table 4:** Chromosome constitution of resistant-pubescent and susceptible-glabrous individuals obtained from self-pollination of BC_12_ 43-chromosome plants Obriy/EF3//Obriy^*12^.

Family	No. of offspring	Susceptible-glabrous plants	Resistant-pubescent plants		% of resistant-pubescent plants with an added chromosome
		Number of plants with chromosome pairing at MI			
		21^II^	21^II^+1^I^	22^II^	
27-94	158	114	42	2	27.9 ± 7.0
193-94	385	313	71	1	18.7 ± 3.9

### Acetocarmine-stained chromosomes in pollen mother cells

Most PMCs of lines O27-2, O27-3 and O193-3 showed 22 bivalents at meiotic metaphase I, indicating cytological stability. While 11.4 ± 4.1% of the cells had 21 bivalents and two univalents, 88.6 ± 4.1% maintained the full 22 bivalents. The presence of the alien chromosome in a disomic addition condition disrupted homologous chromosome pairing, resulting in aneuploidy. The result was that approximately 10% of the progeny from 44-chromosome plants resulted in aneuploids, mostly with 43 chromosomes. This disruption was seen at metaphase I as increased univalents and open (rod) bivalents. On average, the disomic addition lines had 0.26 ± 0.10 univalents and 1.75 ± 0.20 rod bivalents per PMC, compared to the recurrent variety Obriy, which had 0.13 ± 0.05 univalents and 1.12 ± 0.11 rod bivalents per cell. The addition lines also had slightly more closed (ring) bivalents, with 20.1 ± 0.2 compared to 19.8 ± 0.1 in Obriy. Although wheat and alien chromosomes couldn’t be distinguished directly in this analysis, a clear reduction in homologous wheat chromosome pairing was observed in aneuploid plants where the *Elymus* chromosome was always univalent (**Figure 4B**). This reduction was likely due to an unfavorable interaction between wheat and *E*. *sibiricus* genes, leading to partial or complete early bivalent splitting (desynapsis), which disrupted chromosome pairing stability. The impact of these genetic interactions was generally mild, but led to a slight increase univalents (on average 0.13 per PMC) and more frequent rod bivalent formations (on average 0.63 per PMC). Univalents were observed in around 11.4 ± 4.1% of PMCs, mainly with two observed per PMC. Only 1.3 ± 1.5% of PMCs contained four univalents, while cells containing more than four univalents were not identified. The recurrent cultivar Obriy, on the other hand, had 21 bivalents in 93.5 ± 1.7% of PMCs. Thus, disomic addition of the *E*. *sibiricus* chromosome, which determines leaf rust resistance and hairiness, to the karyotype of the cultivar Obriy most significantly negatively impacted homologous chromosome pairing.

### Fluorescence *in situ* hybridization analysis of addition lines

**Figure 5:**
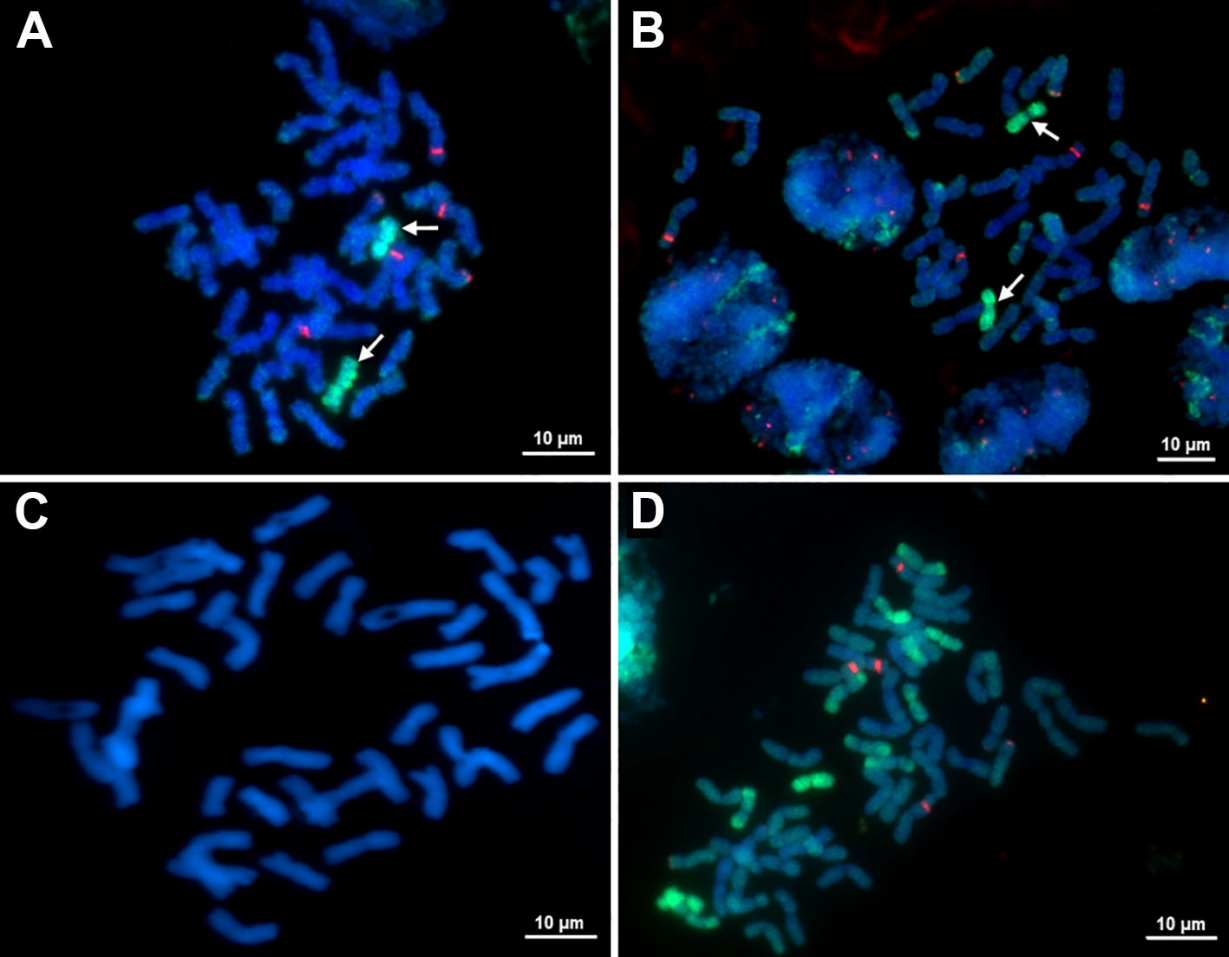
Metaphase chromosomes of **(A)** O27-2 BC_8_F_6_ disomic additional lines (2*n* = 44), **(B)** O27-3 BC_12_F_6_ disomic additional lines 2*n* = 44), **(C)** Mitotic metaphase chromosomes of the recurrent parent cultivar Obriy (2*n* = 42) stained with DAPI, and **(D)** amphiploid EF3 (2*n* = 56). Green fluorescence indicates hybridization of *E. sibiricus* genomic DNA, red fluorescence indicates hybridization of 25S rDNA.

Phenotypic analysis of the BC_12_F_6_ (O27-2) and BC_12_F_6_ (O27-3 and O193-3) stable disomic lines showed the presence of *E*. *sibiricus* chromosome, as indicated by two notable traits: necrotic resistance to leaf rust and specific pubescence of the leaf blades. FISH analysis was used to confirm the presence of genetic material from *E*. *sibiricus* at cytogenetic level. For analysis of partial amphiploid EF3 and all disomic lines, this technique used the total genomic DNA of *E*. *sibiricus*, which is fluorescent in green, and 25S ribosomal DNA (rDNA) probes, which are fluorescent in red. The results indicated presence of *E*. *sibiricus* genetic material in all investigated lines and parental forms (**Figure 5**). Each line contained 44 chromosomes, 42 of which were wheat chromosomes and two of which were *E*. *sibiricus* chromosomes (**Figures 5A–C**). Application of the 25S rDNA probe revealed the typical wheat distribution of 45S rDNA loci on the 1B and 6B satellite chromosomes (marked by red spots) and the 5D chromosomes (**Figure 5D**). The additional chromosomes in the disomic addition lines did not carry 45SrDNA loci and appeared submetacentric (**Figures 5A, B**), further substantiating their alien origins.

### Molecular markers specific to the alien additional chromosomes

**Figure 6:**
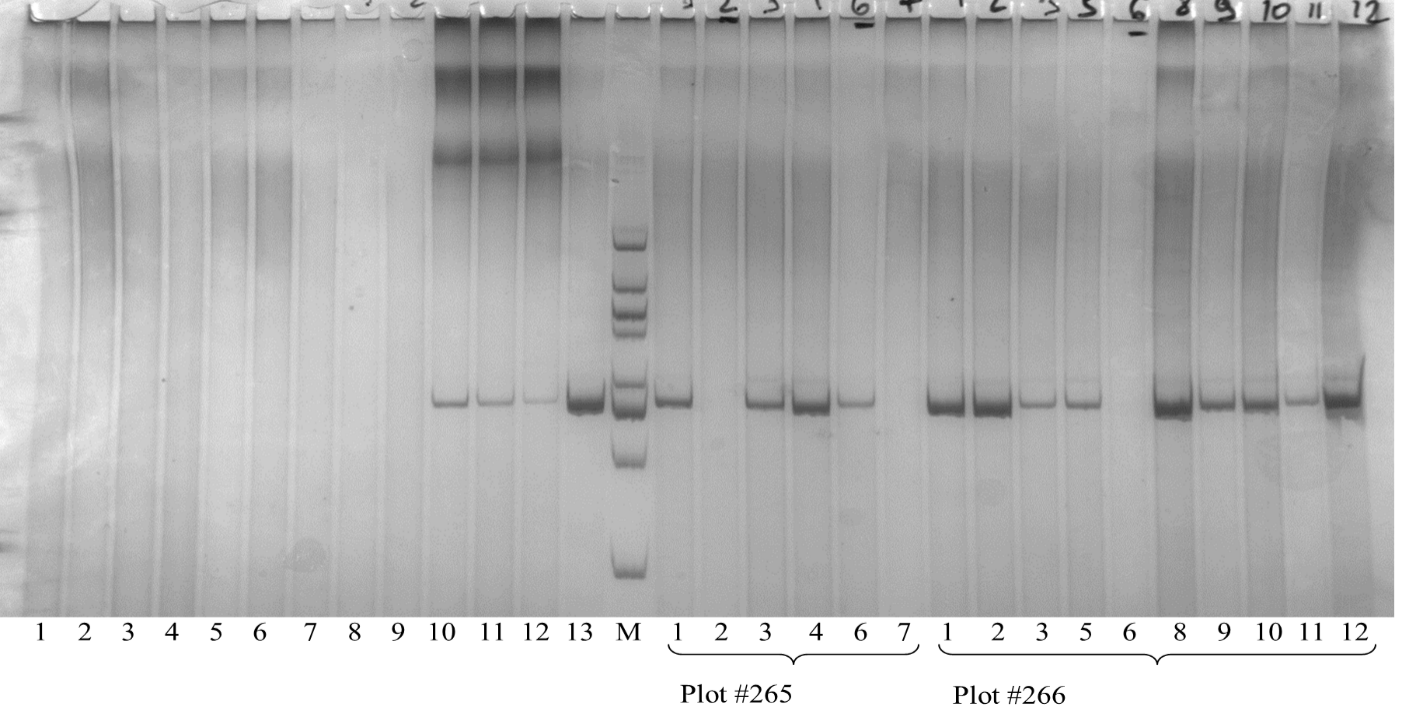
DNA amplification products (500 bp) of *Elymus* species and disomic addition lines at the microsatellite locus *Sr24/Lr24#12*. M – molecular weight marker 20 bp ladder; 1-3 – *E. canadensis*; 4-6 – *E. dahuricus*; 7-9 – *E. sibiricus*; 10-12 – amphiploid EF3; 13 – Agent (T3DS.3DL-3Ae # 1, cultivar-carrier of *Lr24*/*Sr24* genes); 1-7 – individual plants from plot #265 (according to **Table 5**); 1-12 – individual plants from plot #266 (line O193-3).

The markers *Xwmc221* (7DL-7Ae#1L) and *Lr29F18* (DL-7Ae#1L·7Ae#1S) were not detected in lines O27-2, O27-3 and O193-3. In contrast, the *Sr24/Lr24#12* marker (3DL-3Ae#1L) with 500 bp size was positively amplified in EF3, O27-2, O27-3 and O193-3, but not in the cultivar Obriy (**Table 5** and **Figure 6**). Hence, the presence of the *Sr24/Lr24#12* marker, which is located on the 3S^t^ chromosome, indicates that the chromosome added from *E*. *sibiricus* in these lines is in fact the 3S^t^ chromosome. These specific markers made it possible to determine the location of the genetic material from *E*. *sibiricus* in the added alien chromosome, enhancing understanding of the genomic composition of these addition lines. It was found that three *Elymus* species from the PBGI-NCSCI collection did not carry the 500 bp locus of the *Sr24/Lr24#12* marker (**Figure 6**). This finding indicates that the 3S^t^ chromosome in the amphiploid EF3 karyotype may have originated from other species or samples within the genus *Elymus* (or *Agropyrum*), unlike *E*. *sibiricus*. Alternatively, it could point to heterogeneity in *E*. *sibiricus*, suggesting its potential role as a diverse alien genome donor species in the amphiploid. The absence of the 500 bp locus in certain plants underscores the complexity and variability of genetic contributions in these hybrid lines, highlighting the necessity of further exploring the genetic composition and origins of the 3S^t^ chromosome in EF3.

**Table 5:** Results obtained using the *Sr24/Lr24#12 * PCR marker to detect the *Lr24* (3S^t^) leaf rust resistance gene from *Th. ponticum* in paternal forms and disomic addition wheat lines.

Cultivar, line, family	Individual plants									
	1	2	3	4	5	6	7	8	9	10
Cv. Obriy (2*n* = 42)	–	–	–	–	–	–	–	–	–	–
EF3 (2*n* = 56)	+	+	+	+	+	+	+	+	+	+
Line C27 (2*n* = 42)^a^	–	–	–	–	–	–	–	–	–	–
Line O27-2 (2*n* = 44)										
35-1/16	+	+	+	+	+	+	+	+	+	+
35-4/16	+	+	+	+	+	+	+	+	+	+
35-5/16	+	+	+	+	+	+	+	+	+	+
35-6/16	+	+	+	+	+	+	+	+	+	+
36-1/16	+	–	+	+	+	+	+	+	+	+
36-2/16	+	+	+	+	–	+	+	+	+	+
Line O27-3 (2*n* = 44)										
2253-4/14	+	+	+	–	+	+	+	+	+	–
337-1/16	+	–	–	+	+	+	+	+	+	+
337-2/16	+	+	+	+	+	+	+	+	+	+
338-1/16	+	+	+	+	+	+	+	+	+	–
Line C193 (2*n* = 42) ^a^	–	–	–	–	–	–	–	–	–	–
Line O193-3 (2*n* = 44)										
265/17	+	–	+	+	+	+	–	+	+	+
266/17	+	+	+	+	+	–	+	+	+	+

a Lines C27 and C193 were developed as control lines after the same BC_12_ (Obriy/EF3//Obriy^*12^), but without the *E. sibiricus* traits of leaf pubescence and leaf rust resistance. +  Presence − or absence of the genes according to corresponding marker alleles.

The karyotype of the specified disomic addition lines contains an additional pair of chromosomes from the S^t^ genome of the tetraploid *E*. *sibiricus* (2*n* = 4*x* = 28, S^t^S^t^HH). We also observed phenotypic differences between disomic addition lines, where leaf rust resistance is characterized by necrosis without stem rust resistance (**Figure 3**, **Table 3**), and cultivars and lines containing the typical *Lr24* gene. Lines O27-2, O27-3, and O193-3 were confirmed to be homozygous for the *E*. *sibiricus* 3S^t^ chromosome and showed high levels of leaf rust resistance (**Table 3**). These lines consistently exhibited strong resistance across multiple growing seasons under natural and artificial leaf rust infection conditions. As no leaf rust resistance genes have been previously reported from *E*. *sibiricus*, this study is the first to successfully transfer and integrate an *E*. *sibiricus* chromosome into the wheat genome that confers leaf rust resistance. This finding opens new avenues for understanding the genetic basis of disease resistance in wheat and highlights the complexity of resistance mechanisms involving multiple genes and alleles.

Some plants in the three-disomic addition line families did not exhibit DNA amplification products at the *Sr24/Lr24#12* marker locus (**Table 5** and **Figure 6**). The absence of these traits cannot be attributed to cross-pollination or contamination, as that would result in plants lacking the marker traits with 43 or 42 chromosomes. We employed a combination of molecular markers, phenotypic traits and cytological analysis to select 44-chromosome plants that exhibited leaf pubescence, resistance to leaf rust and the presence of a 500 bp amplicon at the *Sr24/Lr24#12* microsatellite marker locus. This rigorous selection process was crucial for maintaining line constancy and ensuring genetic material integrity in subsequent generations. A focus on these specific characteristics led to isolation and propagation of plants retaining the desired phenotypic characteristics, confirming the presence of the target genetic segment and contributing significantly to the understanding and development of wheat varieties resistant to leaf rust.

### Agronomic performance

Lines containing the *E*. *sibiricus* 3S^t^ chromosome with *Lr^e^* (resistance to leaf rust) and *Hl^e^* (leaf hairiness) genes were found to exhibit significantly lower fertility (averaging 46.6 ± 2.6 grains per main spike) compared with the recurrent cultivar Obriy (66.4 ± 2.9 grains per main spike). The chromosome with *Lr^e^* and *Hl^e^* genes from *E*. *sibiricus*, associated with leaf rust resistance and increased hairy leaves, may contribute to the reduced fertility observed in these addition lines. The introgressed *E*. *sibiricus* chromosome lowered seed set and negatively affected traits such as plant height, productive tillering, spikelet count in the main spike, main spike density and thousand kernel weight, which varied annually (**Table 6**). Other characteristics, such as heading date, number of seeds per spikelet and sedimentation values, did not differ significantly (**Table 6**). Only the protein content increased in these lines, with O27-3 showing 15.8% compared to 13.4% in Obriy (**Table 6**). Disomic-addition line O27-3 displayed shorter growth, reduced spike density (310/m^2^) and lower grain yield = (2.86 t/ha) compared with the recurrent parent (**Table 6**).

**Table 6:** Average values of traits characterizing the development of plants of Obriy, O27-2, O27-3 and *Elytricum fertile* (EF3) lines in the period 2022-2023.

Trait	Line				*LSD_0.05_
	Оbriy 2*n* = 42	О27-2 2*n* = 44	О27-3 2*n* = 44	EF3 2*n* = 56	
Grain yield, t/ha	6.61	–	2.86	–	0.97
Number of spikes/m^2^	502	–	310	–	55.3
Plant height, cm	67.5	66.7	43.2	128.1	4.7
Main spike length, cm	9.8	9.7	7.9	15.8	0.8
Number of spikelets in the main spike	20.8	17.1	15.2	19.5	1.1
Main spike density	20.3	16.8	17.7	11.8	1.0
Number of kernels in the main spike	66.4	51.8	41.3	42.2	6.0
Weight of kernels from the main spike, g	1.97	1.51	1.13	1.19	0.24
Thousand kernel weight, g	26.2	24.5	22.6	25.3	1.7
Protein content, %	13.4	15.1	15.8	17.0	1.1

– No data.

*The LSD_0.05_ (Least Significant Difference) value represents the minimum difference required between two means for that difference to be considered statistically significant at a 95% confidence level.

## Discussion

In this study, three wheat-*E*. *sibiricus* disomic addition lines (O27-2, O27-3 and O193-3) were successfully identified. They were all based on *E*. *sibiricus* chromosome (S^t^), as confirmed by leaf rust field evaluations, molecular marker assays and cytogenetic analysis (GISH, FISH and acetocarmine staining). The process involved either eight (O27-2) or twelve (O27-3 and O193-3) backcross generations, allowing for the gradual restoration of the recurrent parent genome (cultivar Obriy) in these addition lines. Each successive backcross increased the proportion of the recurrent parent’s genetic material while retaining the *E*. *sibiricus* 3S^t^ chromosome. This ensured that the desired alien chromosome segment carrying leaf rust resistance was retained, while minimizing unwanted genetic material from the donor species, enhancing wheat’s genetic integrity. Evaluation of EF3 and its derived lines O27-2, O27-3, and O193-3, all carrying the *E*. *sibiricus* 3St chromosome with leaf rust resistance gene(s), showed effectiveness against the local Ukrainian leaf rust population. Since no previous studies have identified leaf rust resistance genes from *E*. *sibiricus*, this study reports the first successful transfer of the 3S^t^ chromosome from *E*. *sibiricus* to wheat. *E*. *sibiricus* is known for its rich gene pool, which can potentially enhance biotic and abiotic stress tolerance in cereal crops like wheat and barley (Zheng et al., 2022; Yu et al., 2022). The GISH/FISH analyses in this study confirmed that the additional alien chromosome in lines O27-2, O27-3 and O193-3 originated from *E*. *sibiricus*.

The presence of the S^t^ genome chromosome in these wheat lines indicates that there is a complex interaction at the genomic level, which might provide an opportunity to develop disease-resistant or stress-tolerant wheat varieties derived from *E*. *sibiricus*. The variation observed in the alien chromosome among different lines also indicates a diversity of genetic potential for wheat breeding. Agronomic performance data indicate that *E*. *sibiricus* is a valuable source of genetic resources, especially leaf rust resistance genes, which can be exploited for wheat genetic improvement. These findings support the potential of *E*. *sibiricus* to contribute desirable traits to wheat breeding programs focused on disease resistance. The rRNA genes were chosen as probe sites because they are highly replicated, occupy distinct chromosomal locations and have high degrees of sequence conservation across plant groups (Liu et al., 2005; Maluszynska et al., 1998). FISH, GISH and classical cytological analysis are powerful and efficient tools for determining the extent of alien chromatin introgression. In this study, *E. sibericus* genomic DNA and 25S rDNA were employed to identify *E*. *sibiricus* chromatin in the wheat genome through GISH/FISH (Hasterok et al., 2002; Maluszynska, 2002; Unfried and Gruendler, 1990). Using multicolor FISH/GISH, novel wheat-*E*. *sibiricus* addition lines exhibiting leaf pubescence and resistance to leaf rust were characterized (**Figure 2**, **Figure 3**). On the alien chromosomes (green) as shown in **Figure 5**, there was no red fluorescence, which indicates the absence of 45S rDNA on this chromosome. Furthermore, some slides displayed “green shadows” along some wheat chromosomes. This observation led to the hypothesis that these might be instances of nonspecific hybridization or due to synteny in the cereal group, although the exact causes remain unclear.

Thus, all lines contain a pair of intact *Elymus* chromosomes in addition to the bread wheat genome. Notably, the identified chromosome (3S^t^) significantly enhanced resistance to leaf rust, although no similar effect was observed for other diseases (**Table 3**). This aligns with previous findings by Cauderon et al. (1973) in development of an addition line with *Th*. *intermedium* chromosome homoeologues for the third group of wheat homoeologues, specifically determining leaf rust resistance and leaf hairiness. The PMCs of lines O27-2, O27-3 and O193-3 displayed 22 bivalents during meiotic metaphase I, which indicates cytological stability. However, it was observed that the introduction of the alien chromosome from *E*. *sibiricus* in a disomic addition condition led to a low frequency (10%) of non-paired chromosomes, resulting in aneuploidy. This resulted in turn in a slight increase in the frequency of rod bivalents (0.63 per PMC) and univalents (0.13 per PMC). In contrast, the recurrent cultivar Obriy typically exhibited 21 bivalents in 93.5 ± 1.7% of PMCs, highlighting the significant impact of this chromosomal addition on homologous chromosome pairing in these lines. Chromosome rearrangements, such as inversions, translocations, fusions and fissions, significantly affect hybrid fitness, chromosome-pairing behavior and pollen fertility (Heslop-Harrison and Schwarzacher, 2011). On the other hand, addition or deletion of heterochromatin does not significantly affect these factors (Rieseberg, 2001). The GISH/FISH and cytological analysis in this study made it possible to distinguish *E*. *sibiricus* chromosomes from those of wheat origin, thus providing a clear understanding of the genetic composition of these disomic addition lines. These findings have important implications for understanding the genetic and cytogenetic relationships between wheat and *E*. *sibiricus*, providing a potential avenue for future breeding and genetic research.

This study successfully identified lines that exhibit pronounced leaf pubescence (hairiness) and resistance to leaf rust at the adult stage. This finding is significant because leaf pubescence could serve as a valuable phenotypic marker for selection in the field. The presence of pseudo-black chaff, associated with the *Sr2*/*Yr30*/*Lr27* resistance genes, and leaf tip necrosis, linked to the *Lr34*/*Yr18*/*Sr57* genes (Rahmatov et al., 2019), are also used as phenotypic markers for field selection at PBGI-NCSCI. Leaf pubescence phenotypes provide a practical and efficient means of identifying and selecting leaf rust-resistant plants for PBGI-NCSCI breeding programs. There is a strong correlation between leaf pubescence and leaf rust resistance gene(s), which indicates good potential for using these markers to rapidly screen large populations in order to develop wheat varieties with enhanced leaf rust resistance. This approach is especially helpful if molecular markers are not readily available or cannot be used in breeding programs.

In early studies, Sears (1956) and Knott (1961) developed alien addition lines that were combined with irradiation or cytogenetic manipulation of pairing control mechanisms and induced translocations to improve wheat genetics (Cauderon et al., 1973; Khush and Brar, 1992; Rey and Prieto, 2017). Among various sources, perennial wild species carrying the S^t^ genome remain valuable for their agronomic performance (Ceoloni et al., 2015; Curwen-McAdams and Jones, 2017). *Agropyron* and *Elymus* species provide important genetic resources for improving disease resistance in wheat (Han et al., 2014; Li and Wang, 2009; Qi et al., 2015). Specifically, *Th*. *ponticum* is known to have contributed the following *Lr* genes to bread wheat: *Lr19* (7DL-7Ae#1L), *Lr24* (3DL-3Ae#1L) and *Lr29* (DL-7Ae#1L·7Ae#1S) (Wang, 2011). The gene *Lr24*, derived from the 3S^t^ chromosome of *Th*. *ponticum*, is known to be linked to *Sr24* (McIntosh et al., 2020). Lines carrying *Lr24* exhibit high resistance to leaf rust and stem rust at the adult stage. Moreover, lines containing *Lr24* exhibit high resistance to leaf rust and stem rust without chlorosis or necrosis on the flag leaves of adult plants (Bolton et al., 2008; McIntosh et al., 1977). We found that lines O27-2, O27-3 and O193-3 exhibited distinct differences in their reaction to leaf rust and stem rust compared with accessions known to possess *Lr24*/*Sr24* resistance genes. Chromosomal rearrangements, such as translocations, inversions, and fusions, can alter chromosome structure and result in the loss of specific marker loci (Przewieslik-Allen et al., 2021). Similar phenomena have been observed in wheat-*Th. ponticum* substitution lines, where certain marker loci fail to amplify due to structural changes in the alien chromosome or instability during integration (Wang et al., 2020).

This study assigned the molecular marker *Sr24/Lr24#* to homologous group 3, specific to the additional chromosomes of the O27-2, O27-3 and O193-3 lines. Therefore, the leaf rust resistance gene in lines O27-2, O27-3 and O193-3 is a novel resistance gene from *E*. *sibiricus*. Chromosome translocation lines are being developed to transfer the leaf rust resistance gene into the bread wheat genome. Further research is required to determine whether the leaf rust resistance gene on the 3S^t^ chromosome is a single dominant gene. The unique molecular marker *Sr24/Lr24#* found on the additional chromosomes of O27-2, O27-3 and O193-3 can also detect and select alien chromosomes or their segments carrying resistance genes to leaf rust. This study broadens the range of valuable genetic resources for discovering novel genes and analyzing genetic diversity in *E*. *sibiricus*, thus providing breeders with additional tools to develop leaf rust-resistant wheat varieties. Current efforts by our research group are concentrating on developing interstitial recombinants with shortened S^t^ fragments from *E*. *sibiricus*, a strategy to enhance wheat genetic foundation by incorporating desirable traits from this wild species. Through this approach, the genetic diversity available for wheat improvement will be expanded and specific resistance genes will be transferred, potentially leading to the development of more robust and resilient wheat varieties.

## Conclusions

By combining the bread wheat cultivar Obriy and a partial wheat-*E*. *sibiricus* octoploid amphiploid, we successfully developed three wheat-*E*. *sibiricus* disomic addition lines, O27-2, O27-3 and O193-3. These lines exhibit distinct leaf hairiness linked to the 3S^t^ chromosome and are resistant to leaf rust, representing a significant achievement in unlocking the potential of this valuable gene(s) for wheat improvement. Molecular marker assays and cytogenetic analyses identified wheat-*E*. *sibiricus* disomic addition lines carrying the 3S^t^ chromosome, which exhibit leaf hairiness and resistance to leaf rust. Our research also encompasses ongoing chromosome engineering endeavors, with a primary focus on reducing the *E*. *sibiricus* segment through *ph1b*-induced homoeologous recombination. This process involves manipulating the *ZIP4* gene at the *Ph1* locus, which regulates homologous chromosome pairing in wheat, thus facilitating recombination between wheat and *E*. *sibiricus* chromosomes (Rey et al., 2017). Furthermore, we have identified leaf hairiness as a valuable phenotypic field marker related to leaf rust resistance. In this study, we confirmed the importance of the *E*. *sibiricus* S^t^ genome as a valuable genetic resource, providing promising solutions to address challenges related to leaf rust in wheat production. Developed lines provide a novel germplasm resource, which offers a promising pathway for incorporating leaf rust resistance genes into wheat improvement efforts.

## Data Availability

The datasets presented in this study are available in online repositories. The names of the repositories and accession numbers are in the article/supplementary material.
